# The Contribution of Resting State Networks to the Study of Cortical Reorganization in MS

**DOI:** 10.1155/2013/857807

**Published:** 2013-10-31

**Authors:** Rosaria Sacco, Simona Bonavita, Fabrizio Esposito, Gioacchino Tedeschi, Antonio Gallo

**Affiliations:** ^1^II Division of Neurology, Second University of Naples, Piazza Miraglia 2, 80138 Naples, Italy; ^2^MRI Center “SUN-FISM,” Second University of Naples and Institute of Diagnosis and Care “Hermitage-Capodimonte,” Piazza Miraglia 2, 80138 Naples, Italy; ^3^Department of Neuroscience, University Federico II, 80131 Naples, Italy

## Abstract

Resting State fMRI (RS-fMRI) represents an emerging and powerful tool to explore brain functional connectivity (FC) changes associated with neurologic disorders. Compared to activation/task-related fMRI, RS-fMRI has the advantages that (i) BOLD fMRI signals are self-generated and independent of subject's performance during the task and (ii) a single dataset is sufficient to extract a set of RS networks (RSNs) that allows to explore whole brain FC. According to these features RS-fMRI appears particularly suitable for the study of FC changes related to multiple sclerosis (MS). In the present review we will first give a brief description of RS-fMRI methodology and then an overview of most relevant studies conducted so far in MS by using this approach. The most interesting results, in particular, regard the default-mode network (DMN), whose FC changes have been correlated with cognitive status of MS patients, and the visual RSN (V-RSN) whose FC changes have been correlated with visual recovery after optic neuritis. The executive control network (ECN), the lateralized frontoparietal network (FPN), and the sensory motor network (SMN) have also been investigated in MS, showing significant FC rearrangements. All together, RS-fMRI studies conducted so far in MS suggest that prominent RS-FC changes can be detected in many RSNs and correlate with clinical and/or structural MRI measures. Future RS-fMRI studies will further clarify the dynamics and clinical impact of RSNs changes in MS.

## 1. Introduction

In the last decade magnetic resonance imaging (MRI) has acquired a central role in the clinical [1] and research setting in multiple sclerosis (MS) [2]. 

The MRI approach to MS includes traditional MRI techniques, that allow to identify focal white matter (WM) lesions and advanced MRI techniques (A-MRI), which allow to further characterize structural and functional changes related to MS.

Among different A-MRI techniques, functional MRI (fMRI) allows to explore the dynamics of cortical functional reorganization associated with MS and its impact on disease evolution [2, 3].

Most of the fMRI studies conducted so far in MS have had an activation paradigm evoked by simple motor tasks, sensory stimulation, cognitive tasks, and so forth. These activation/task-related studies have shown significant and consistent functional changes at the level of relevant cortical networks in MS patients when compared to healthy controls (HCs). Such findings have been further supported by correlations with behavioral measures as well as markers of brain tissue injury, such as T2 lesion load (T2-LL) [4, 5] and A-MRI-derived metrics [6–8].

Task-related fMRI studies have, however, at least one major inherent limitation represented by difficulties in interpretation of results due to large intersubject variability in task performance, a problem that is even more pronounced in disabled people.

Such limitation of task-related fMRI studies can be overcome by a more recent approach, acquiring fMRI data during resting state (RS) conditions, that is, with subjects awake, but relaxed and not involved in any task. In this setting, an appropriate processing of the data allows the extraction of a number of so-called resting state networks (RSNs) that mostly reflect well-characterized functionally relevant networks. 

On this background, the RS-fMRI approach has soon appeared particularly suitable to explore large-scale functional changes taking place in a clinical and pathological heterogeneous disease such as MS.

In the present review, we will first describe RS-fMRI methodology and then present and discuss most relevant RS-fMRI results obtained so far in MS. 

## 2. Resting State-fMRI: Background and Description of Principal Resting State Networks

Recent advances in fMRI have provided new tools to measure functional interactions between brain regions, focusing on the examination of functional connectivity (FC) in the human brain at rest. FC can be defined as the temporal dependence of neuronal activity patterns of brain anatomically- distinct regions [9]. 

RS-FC can be assessed noninvasively *in vivo* by analyzing the spatiotemporal distribution of spontaneous low-frequency (<0.1 Hz) BOLD (blood-oxygen-level dependent) fMRI signals fluctuations during resting conditions throughout the brain [9].

Multiple analysis techniques have been applied to RS-fMRI dataset. The most commonly used ones are the region-of-interest (ROI) based analysis and the independent component analysis (ICA) [10]. While the first is based on the time-course extraction of the BOLD signal from a predefined ROI and subsequent identification of brain regions displaying overlapping BOLD signal fluctuations (i.e., functionally connected to predefined ROI) [11], the ICA approach is a statistical-based technique that uses a mathematical algorithm to decompose a set of signals into independent components which are spatial maps associated with the time courses of the signal sources. Each component may in fact be interpreted as a network that encompasses brain regions sharing the similar BOLD fluctuation across time. RS-fMRI studies applying ICA have shown a high level of consistency but sometimes the extracted components are difficult to interpret [12]. 

A fast growing body of neuroimaging studies supports the notion that RS BOLD fluctuations of cortical and subcortical regions originate, at least in part, from spontaneous neuronal activity and that the observed temporal correlation between fMRI time series of anatomically separated regions is reflecting a level of ongoing FC between brain regions during RS allowing to identify the so-called resting state networks (RSNs) [11]. 

The most consistent RSNs are the default mode network (DMN), the sensorimotor network (SMN), the visual (V-RSN) and auditory (A-RSN) networks, the executive control network (ECN), the lateralized frontoparietal network (FPN), and the temporoparietal network (TPN) [13]. While the SMN and the visual and auditory networks involve cortical regions normally engaged in sensorimotor, visual, and auditory processes, the DMN and the FPN are the RSNs most relevant for cognition.

The DMN is the most and best studied cognition-related RSN. It involves the precuneus/posterior cingulate cortex (PCC), the lateral parietal cortex (LPC), and the medial prefrontal cortex/anterior cingulate cortex (ACC) [14, 15]. 

The DMN is highly activated during RS and deactivated during the execution of working memory and attention cognitive tasks [14–16]. According to these prerogatives, DMN FC can be investigated using different experimental designs. The most commonly-used paradigm is based on acquisition of the whole RS-fMRI dataset while patients are lying in the scanner awake, relaxed, and with their eyes closed. Alternatively, DMN can be evaluated during resting periods inserted as a part of a block design during a cognitive task. Finally, DMN can be extracted as a task-negative network that is strongly deactivated during cognitive tasks.

Intrinsic DMN FC has been found to change in physiological and preclinical conditions such as normal aging [17] and APOE-epsilon4 status [18]. Moreover, a growing number of studies suggest that DMN might be a potential surrogate marker of disease in the following neurological conditions: mild cognitive impairment and Alzheimer's disease [19–22]; parkinsonian syndromes [23, 24]; motor neuron disease [25–28]; clinically isolated syndrome (CIS), and MS [29–36]. Finally, very recent evidences in MS suggest that DMN FC might be significantly modulated by cognitive reserve [37] and cognitive rehabilitation [38, 39].

The SMN includes the precentral gyrus, the postcentral gyrus, and the supplementary motor area that are all normally involved in motor tasks [40, 41].

Up to three V-RSNs have been reported in scientific literature. Most commonly it is possible to identify a primary V-RSN (pV-RSN), including calcarine/striatal and pericalcarine/peristriatal regions and a secondary V-RSN (sV-RSN), consisting of extrastriatal occipital regions, as well as occipitotemporal and occipitoparietal gyri [42]. A clear remodeling of intrinsic V-RSN connectivity has been observed in early blind subjects [43], Leber's hereditary optic neuropathy [44], MS [32, 45], and HCs after alcohol intake [46]. The A-RSN involves the superior temporal gyrus, Heschl's gyrus, the insula, and the postcentral gyrus [10, 11].

The ECN, which is constituted by the medial frontal gyrus, superior frontal gyrus, and the ACC, is involved in executive functions, such as control processes and working memory [47].

The FPN is represented by two distinct but specular components on the right and left hemisphere. The FPN involves the inferior frontal gyrus, the medial frontal gyrus, the precuneus, the inferior parietal, and the angular gyrus [10]. This RSN has been associated with different functions such as memory, language, attention, and visual processing [10].

Finally, the TPN, which includes the inferior frontal gyrus, medial temporal gyrus, superior temporal gyrus, and the angular gyrus has shown to be involved in language processing [10].

## 3. Resting State-fMRI: Other Methodological Aspects Relevant to the Study of RSNs

In order to better interpret RS-fMRI data in pathological conditions such as MS, GM, and WM structural damage should be assessed at the same time. Indeed, without a precise assessment of GM damage—that in MS, for example, can be represented by both cortical lesions and focal/diffuse cortical atrophy—it would be difficult to interpret the observed RS-FC changes as driven by colocalized structural GM damage or “purely” functional changes. The same apply to WM damage, which is particularly widespread and severe in MS and can cause RSN changes secondary to structural disconnection between RSN nodes. 

Only few RS-fMRI studies conducted in MS have explored the relationship between structural brain damage and RS-FC changes [29–31, 45, 48]. Interestingly, all these studies have shown that (i) RS-FC changes do not colocalize with GM damage [31, 45], and (ii) damage of specific WM structures can have a significant impact on RS-FC changes [30, 48].

Future studies will have to further integrate structural and RS-FC data in order to better characterize the interplay between mechanisms of (structural) damage and mechanisms of (functional) neuroplasticity.

## 4. RS-fMRI Studies in MS

### 4.1. Default Mode Network

#### 4.1.1. fMRI Data Acquisition Paradigm: Resting Conditions without Any Active Tasks

The integrity of DMN has been evaluated in patients with different forms of MS. In a first study, Roosendaal et al. investigated the main RSNs in 14 CIS patients, 31 relapsing-remitting MS (RRMS) patients, and 41 HCs. CIS patients showed an increased FC in a number of RSNs, including the posterior components of the DMN, when compared to HCs and RRMS patients [29]. This result was interpreted by the authors as suggestive of an early cortical reorganization taking place in the very first stages of MS. Results coming from RRMS patients, contrariwise, did not show any significant change (including disconnection) in all studied RSNs, when compared to HCs [29]. This latter result might reflect the fact that RRMS population was characterized by a relatively mild disease with short disease duration, low EDSS, and mild cognitive deficits. Taken together, the abovementioned RS-fMRI results, integrated with structural data (showing a lower damage in CIS patients when compared to RRMS patients), support the hypothesis that cortical reorganization of RSNs might play an early but limited compensatory role in MS [29].

Similar findings were obtained by Faivre et al. who reported a general increase in the FC of many RSNs, including the DMN, in a group (*n* = 13) of very early RRMS compared to HCs (*n* = 14) [32]. 

In another study conducted by Rocca et al., the authors explored the DMN in HCs, secondary progressive (SP) and primary progressive (PP) MS patients [30]. The between-group analysis showed a significant difference in DMN connectivity (with reduced connectivity in MS subjects) at the level of medial prefrontal cortex (mPFC), left precentral gyrus (PcG), and anterior cingulate cortex (ACC). At post hoc analysis, compared to controls, patients with SPMS had reduced activity in the mPFC and PcG, while patients with PPMS had reduced activity in the PcG and the ACC. Patients with SPMS compared to those with PPMS had increased ACC activity. Notably, FC derangement of the ACC was more pronounced in cognitively impaired patients—independently of MS phenotype—and correlated with the degree of cognitive impairment as well as the severity of structural damage of relevant WM bundles [30].

Bonavita et al. explored the DMN in 36 (18 cognitively preserved [CP] and 18 cognitively impaired [CI]) RRMS patients and 18 HCs [31]. When compared to HCs, the whole group of MS patients showed (i) a reduction of FC at the level of the ACC and the central/midline portion of the PCC; (ii) a significant increase in FC at the level of the peripheral/lateral portions of the PCC as well as LPC, bilaterally. Notably, the increased FC in the posterior components of the DMN was more pronounced in CP RRMS patients. These results, therefore, besides confirming the anterior dysfunction of the DMN in MS (as already reported by Rocca et al. [30]), seem also to suggest that a more favorable MS course (i.e., RRMS versus SPMS and PPMS) and a preserved cognitive function might be linked to reinforcement of FC in the posterior components of DMN [31]. 

At this point, it should be also mentioned that some evidences support the notion that reinforced RSNs can be also associated with a worse clinical phenotype. In the study from Hawellek et al. [33], indeed, the authors were able to show that a stronger DMN connectivity was associated with poorer cognitive performances. 

Therefore, while the compensatory hypothesis (i.e., stronger RSN FC = better clinical status) is still prevailing, a more recent view suggest that—at least in MS—the relationship between RSNs changes and clinical status is not straightforward and might be strongly influenced by individual characteristics of each patient, (see, e.g., genetic background, cognitive reserve, disease duration/phenotype and, quality/quantity of tissue damage).

#### 4.1.2. fMRI Data Acquisition Paradigm: Resting Conditions Interleaved with Active Conditions (Cognitive Task)

The ACC, a DMN component, is known to play a crucial role in higher executive functions. Based on this premise and knowing that MS patients often present a dysexecutive syndrome, Loitfelder et al. [34] decided to investigate the RS-FC of the ACC by studying the rest phases of a stimulus-response discrimination (Go-No Go) task with a block design. Using this data-analysis approach and the ACC as a seed region, the authors were able to show an increased FC between ACC and the left angular gyrus, left PCC, and right postcentral gyrus in MS patients (*n* = 31) when compared to HC (*n* = 31). Better cognitive performances in MS patients were associated with an increased FC with the cerebellum, middle temporal gyrus, occipital pole, and the angular gyrus. The results of this work provided further evidence for adaptive changes in RS-FC in MS patients compared to HCs in a relevant cognitive-related network. Nevertheless, this study had some limitations. The first is represented by the need to define an a priori hypothesis regarding the seed region from where to extract the RSN. Secondly, a residual effect of the interleaved active conditions on the defined frames of relative rest cannot be fully excluded [34]. 

As mentioned above in the paragraph on background and description of principal RSN, DMN can be also extracted and assessed as a task-negative network that is deactivated during cognitive tasks.

Forn et al., using this approach, investigated abnormalities of activation/deactivation patterns in the functional networks related to “task-positive” and “task-negative” events during the execution of the Symbol Digit Modalities Test (SDMT) in CP, CIS, and HCs [35]. The label “task positive” designated the brain networks activated during the execution of a specific task. Conversely, “task negative” designated neural activity that was turned on at rest and reduced the activation when individuals were engaged in any specific cognitive operation. “Task-positive” responses to task execution and “task-negative” activity of the DMN were compared between groups. Compared to HC, CIS patients exhibited an enhanced deactivation of the “task-negative” network at the level of the PCC, whereas no differences between groups were found when the patterns of “task-positive” events were compared. Based on these results the authors concluded that CIS patients show an enhanced pattern of brain deactivations during cognitive performances, which might contribute to their normal neuropsychological status [35].

Finally, using a similar RS-fMRI data acquisition and analysis approach, Sumowski et al. investigated (i) DMN deactivation during sustained attention relative to rest as a neurophysiologic biomarker of memory functioning in MS [36]; (ii) the relationship between cerebral activity within the brain's DMN, intellectual enrichment and cognitive performance during a working memory task (i.e. N-Back) in a sample of MS patients [37]. After evaluating brain atrophy as well, the results of both studies suggested that DMN integrity is strictly related to intellectual enrichment [37] as well as a better predictor of memory integrity than brain atrophy [36].

DMN studies conducted with alternative fMRI data acquisition/analysis protocols, therefore, substantially overlap those conducted with the standard protocol (i.e., during constant resting conditions) showing the relevance of DMN changes to cognitive status in MS.

#### 4.1.3. DMN and Cognitive Rehabilitation

Very recently, RS-fMRI studies exploring cognitive-related networks, including DMN, have been conducted with different experimental designs to monitor cognitive rehabilitation treatments in MS patients [38, 39]. Preliminary results, coming from the two works conducted so far, seem to suggest that cognitive rehabilitation is associated with improved neuropsychological performances as well as a stronger RS-FC of cognitive-relevant RSNs, including DMN [38, 39]. 

Future investigations will have to further assess and clarify the potential application of this new approach to the efficacy evaluation of different cognitive rehabilitation programs. 

### 4.2. Visual Resting State Network

Anterior and posterior visual pathways are often affected by MS pathology. fMRI studies with visual stimuli have often been used to explore neuroplasticity in the visual cortex following optic neuritis (ON) [49–51]. Such investigations have consistently shown relevant and dynamic functional changes taking place in the primary and secondary visual areas during ON. This approach, however, is strongly limited by the fact that the measured signal mostly reflects the degree of anterior visual pathway damage—that is, the signal that reach the cortex—rather than intrinsic activity of visual areas. Moreover, since the anterior visual pathway damage can be highly variable between subjects with ON, this determines a large intersubject variability which strongly undermine results analysis and interpretation.

On this background, RS-fMRI certainly represents a promising approach to explore spontaneous (self-generated) activity of cortical visual areas in a way that is independent of both anterior and posterior visual pathways involvement.

In the study by Roosendaal et al., where all main RSNs were explored, the authors did not find any significant change in V-RSN in both CIS and RRMS patients when compared to each other and HCs [29]. Nonetheless, it should be considered that the general and explorative nature of this study—with no clinical or instrumental evaluation of the visual pathways as well as detailed anamnestic data on previous ON—made it unsuitable for subgroup analyses aimed at exploring V-RSN changes related to visual pathways damage and/or ON [29].

In a similar design study by Faivre et al., RRMS patients with recent disease onset showed, compared to HCs, an increased FC inside multiple regions of the V-RSN (i.e., lingual gyrus, left middle occipital gyrus, and left cuneus), thus suggesting a possible compensatory effect of such changes [32]. 

We recently investigated a population of 30 RRMS patients (16 without [nON-MS] and 14 with [ON-MS] previous ON) and 15 HCs [45] in order to specifically explore the effect of previous ON on intrinsic V-RSN connectivity. To this purpose all subjects underwent a 3TMRI including RS-fMRI data acquisition, a neurological examination and a thorough ophthalmologic evaluation including the assessment of visual acuity as well as the measurement of retinal nerve fiber layer (RNFL) thickness [45]. When the entire group of RRMS patients was compared to HCs, a weakened V-RSN connectivity was found in RRMS patients at the level of inferior peristriate cortices (along the fusiform gyri), bilaterally. The subsequent comparison of ON-MS versus nON-MS patients showed a spot of stronger FC in the right extrastriate cortex (along the middle occipital gyrus) as well as a spot of significant reduced FC in the right inferior peristriate cortex, in ON-MS patients. Notably, all detected V-RSN changes did not colocalize with regional GM atrophy. Taken together our results suggest that (i) normal-sighted RRMS patients, independently of previous ON, have a significant and diffuse functional disconnection in the V-RSN; (ii) RRMS patients recovered from a previous ON show a complex reorganization of the V-RSN, including increased FC at the level of extrastriate visual areas which might play a role in visual recovery [45].

Future studies with a longitudinal design will further clarify the dynamics of V-RSN changes associated with ON.

### 4.3. Other Resting State Networks

In the above-mentioned study by Roosendaal et al., patients with CIS, compared to HCs, showed a significantly higher synchronization in the ECN (left medial prefrontal cortex), FPN (bilateral precuneus) and SMN (premotor, supplementary motor, and sensory cortices) [29]. When compared to RRMS, patient with CIS displayed a significantly higher synchronization in the precuneus as well as the left and right FPN. Finally, RRMS patients lacked to show any significant FC change in the explored RSNs when compared to HCs [29].

Similar results were reached in the Faivre et al. work investigating RSNs in early RRMS patients and HCs [32]. In this study seven of the eight RSNs analyzed, including the SMN and FPN, showed an increased FC in diseased subjects. Notably, the authors were also able to find an inverse correlation between the increased activity of RSNs and disability scores, as measured by MSFC [32].

The results of the above-mentioned studies [29, 32] further support the hypothesis that an increased RSN FC might help compensating—at least for a limited period of time and up to a certain level—the widespread brain tissue damage caused by MS.

Finally, using a newer approach, Richiardi et al. recently studied a group of minimally disabled MS patients and HCs, in order to identify which pattern of RS-FC changes is more specific for MS [52]. The authors were able to show that the most discriminative pattern of RS-FC changes related to MS was localized in subcortical and frontoparietotemporal regions [52]. Needless to say that a further validation of such results might help developing new tools for diagnosis and management of MS patients.

## 5. Conclusions

RS-fMRI represents an emerging powerful tool to explore whole-brain FC changes associated with MS.

RS-fMRI studies conducted so far in MS have shown relevant FC changes in all main RSNs, often reporting significant correlations with validated clinical (i.e., physical and cognitive disability scores) and paraclinical (i.e., MRI-derived) measures [29–39, 45, 48, 52]. 

Based on available data, RSNs rearrangements might be interpreted, at least in the early phases of MS, as adaptive neuroplastic changes compensating for CNS tissue damage.

The years ahead will be crucial for further understanding the real potential and application of this rapidly evolving neuroimaging technique to the study of pathophysiology as well as diagnosis and monitoring of MS. 
